# A case of a rare type of cancer: Anal squamous cell carcinoma in a patient without significant risk factors

**DOI:** 10.5339/qmj.2024.7

**Published:** 2024-02-15

**Authors:** Kevan English, Mercedes Erpelding, Sandra Kaldas, Sabrine Semoin

**Affiliations:** ^1^Department of Surgery, St. George’s University School of Medicine, Saint George, Grenada Email: kenglish@sgu.edu ORCID iD: 0009-0006-8893-5696; ^2^Department of Surgery, Ross University School of Medicine, Bridgetown, Barbados; ^3^Department of Surgery, Jackson North Medical Center, Miami, Florida, USA

**Keywords:** Anal cancer, squamous cell cancer, anal canal, adenocarcinoma, human papillomavirus

## Abstract

Introduction: Anal carcinoma is a relatively uncommon tumor that accounts for less than 2% of large bowel malignancies and approximately 1-6% of anorectal tumors. Most anal cancers originate in the mucosa between the anorectal junction and the anal verge. Risk factors for anal carcinoma include human papillomavirus (HPV), immunosuppression, older age, female gender, and smoking. Approximately 85% of anal cancers are squamous cell carcinoma, and the pathophysiology is believed to be linked to HPV-related inflammation, leading to dysplasia and progression to cancer.

Case Presentation: We present the case of a 65-year-old woman who sought medical attention at the emergency department (ED) due to rectal pain and concurrent rectal lesions persisting for the past three months. Before admission, she reported abdominal discomfort and constipation for 2-3 months, during which she took laxatives for relief. Laboratory findings in the ED were significant for anemia and leukocytosis, with all other values within normal limits. Blood tests, including antibodies for HPV and human immunodeficiency virus, were negative. A computed tomography scan of the abdomen and pelvis was largely unremarkable.

On physical examination, perianal lesions with heaped-up edges were observed. A punch biopsy was subsequently performed, revealing squamous cell carcinoma (SCC). About three weeks following discharge, after one week of admission to the general medicine ward, the patient started chemoradiation therapy and reported some improvement in her symptoms. Seven weeks later, she was in remission.

Discussion: Squamous cell carcinoma (SCC) of the anus, a rare disease entity, is often a slow and progressive malignancy. The length of time for patients to become symptomatic, in combination with its mimicking clinical presentation to common gastrointestinal tract diseases and its rarity, makes diagnosis challenging. Additionally, a patient lacking traditional risk factors for anal cancer, such as HPV and smoking, may further complicate diagnosis, treatment, and quality of life.

Conclusion: This case report emphasizes the pathogenesis and the similarities in clinical presentation of anal cancer to mild diseases, which may lead to a delay in diagnosis. Patients with anal carcinoma often delay seeking medical care, which is anecdotal in relation to the considerable overlap in symptoms of benign diseases such as hemorrhoids. Therefore, patients with “hemorrhoid” complaints, rectal bleeding, or rectal mass should warrant further physical examination and prompt referral to a gastroenterologist or a colorectal surgeon for additional evaluation.

## Introduction

Anal carcinoma is a rare type of tumor that comprises less than 3% of cancers of the intestines.^1^ In the general population, anal carcinoma is unusual, with an incidence rate of approximately 1.7 per 100,000 persons in the United States alone, according to data from the National Cancer Institute’s Surveillance, Epidemiology, and End Results Program (SEER) in 2020.^2^ The vast majority of anal cancers are due to squamous cell carcinoma (SCC), with only 10% attributed to adenocarcinoma and 5% related to rare tumors such as melanoma, basal cell carcinoma (BCC), and small cell carcinoma.^3^ Anal cancer typically originates at the anal squamocolumnar junction and arises from precancerous lesions termed anal intraepithelial neoplasia (AIN), also called squamous intraepithelial lesions (SILs). The presence of SILs in high-risk patient groups, such as human immunodeficiency virus (HIV) positive patients, is an indication for frequent surveillance via high-resolution anoscopy.^4^ The classic presentation of anal cancer includes rectal bleeding, discomfort, itching, discharge from the anus, and changes in bowel habits.^5^ Some patients may also have pain and/or a mass sensation.^6^ The constellation of symptoms experienced by patients is commonly confused with the general symptoms of hemorrhoids or anal fissures. Thus, as a result, patients tend to delay seeking medical advice, and the unclear clinical picture poses a challenge to diagnosis.^6,7^ Patients may also clinically appear asymptomatic, leading to diagnostic and treatment problems.^8^ Therefore, physicians should retain a high index of clinical suspicion for anal carcinoma in patients with symptoms resembling benign diseases and other generally benign medical conditions. We present a case of a rare type of cancer and discuss its pathogenesis, epidemiology, and diagnostic challenges in detail. Written informed consent was obtained from the patient regarding the publication of this article and the associated image.

## Case Presentation

A 65-year-old woman arrived at the emergency department (ED) reporting a three-month history of rectal pain and bleeding. Before admission, she was seen by her primary care physician (PCP) for abdominal discomfort, itching, and constipation over the preceding 2-3 months, during which she used therapeutic laxatives. She was given a presumptive diagnosis of hemorrhoids. The patient was seen in an urgent care center one day before the ED visit for urinary tract infection symptoms, during which she complained of a new rectal lesion. Upon further evaluation by her PCP, she was sent to the ED. On presentation, vital signs were within normal limits. Physical examination showed a well-appearing woman in no acute distress. Lungs were bilaterally clear to auscultation with a soft abdomen; non-tender in all four quadrants. Extremities were symmetric with no joint swelling or pitting edema. The patient’s medical history revealed a pre-existing condition of hypertension, while her social history indicated occasional alcohol consumption, with no tobacco or substance use. Laboratory test results from the ED revealed anemia (hemoglobin 9.2 g/dL) and leukocytosis (12.6 WBCs/L). All other values, such as liver function test, electrolytes, and coagulation profile, were within normal limits. No abnormalities resembling leukemia, lymphoma, or bleeding disorders were found on examination of the blood. Immuno-examination results were negative for HIV and human papillomavirus (HPV) antibodies. A computed tomography (CT) scan of the abdomen and pelvis was performed, which revealed colonic diverticulosis and multiple uterine fibroids but no acute intra-abdominal/pelvic pathology.

**Figure 1.**
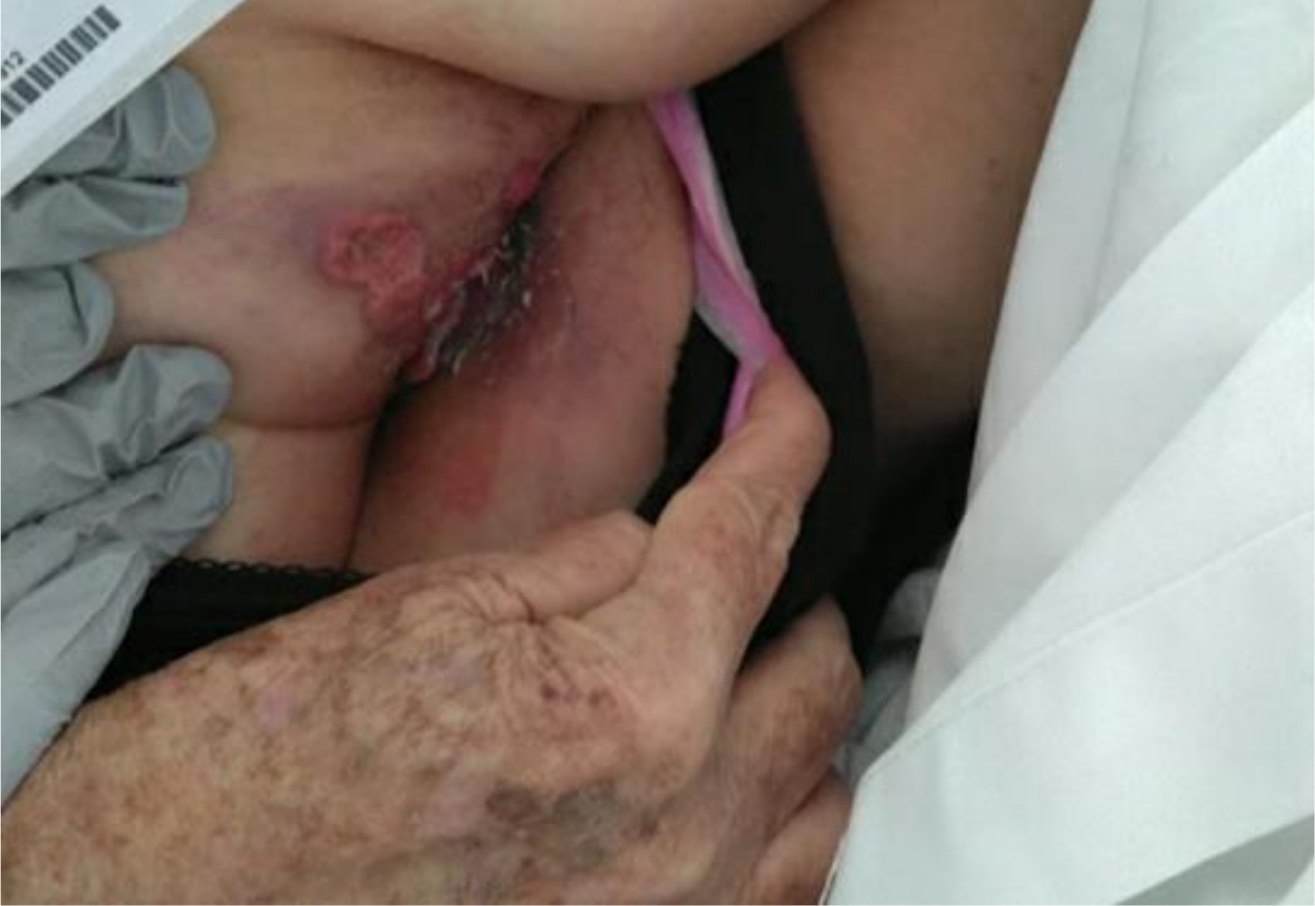
Image showing large exophytic friable lesions with heaped-up edges localized around the anal canal, representing anal squamous cell carcinoma.

On inspection of the anus, friable perianal lesions with heaped-up edges were noted (Figure 1). A punch biopsy (0.4 x 0.3 x 0.3 cm) was performed after that to confirm the diagnosis, which revealed well-differentiated SCC as characterized by the formation of keratin and the presence of intercellular bridges on histology. A positron emission tomography (PET) scan to detect disease metastasis was subsequently performed, which was negative. The patient was discharged from the hospital one week after being admitted to the general medicine ward due to rectal pain and bleeding, which had occurred immediately after her visit to the ED. At her three-week outpatient follow-up, she started the first of two chemoradiation cycles (5-fluorouracil and mitomycin), totaling six weeks. The patient reported mild improvements in her symptoms, citing no rectal pain but occasional bleeding. At her 6-week follow-up, after she completed treatment, she complained of diarrhea and fatigue, but rectal pain and bleeding were absent. One month later, the patient was in complete remission and was referred to a medical oncologist for further follow-ups.

## Discussion

While most anal malignancies are related to HPV infection, it is relatively uncommon for most patients with this infection to acquire anal cancer.^9^ Anal tumors are linked to various subtypes of HPV infection, most notably HPV-16.^10^ Another subtype, HPV-18, is also found in some tumors, but it is far less common than HPV-16. Subtypes 6 and 11 are much less likely to become malignant and are frequently found in anogenital warts.^11^ Verruca vulgaris or common warts are typically linked to low-risk HPV subtypes 1, 2, 4, and 7, with occasional high-risk subtypes (16 and 18) as a cause.^12^ The most common type of anal cancer is SCC. Approximately 90% of anal SCC tumor samples are HPV-positive, varying in incidence according to geographic location, with a higher proportion observed in Western Europe, Northern America, and Oceania.^9,13^ Despite the common association of HPV with anal cancer, our patient had a negative test for the infection, adding a peculiar finding to the case.

Additionally, other risk factors, such as HIV and smoking, were absent from the patient’s history. The combination of anal SCC being rare, as mentioned earlier, and its apparent unclear etiology and pathogenesis in many cases complicated the recognition and prompt diagnosis by the physician. The presenting symptoms also posed difficulty in diagnosis. 

There are two different types of tumors arising in the anal canal region: mucosa-derived tumors, termed anal canal tumors, and skin-derived cancers within the squamous mucocutaneous junction, termed perianal cancers.^14^ In contrast to anal carcinomas, perianal cancers are staged and treated as skin cancer. The staging of anal carcinomas should be determined according to the American Joint Committee on Cancer or AJCC.^15^ Metastatic spread to the lymph nodes is a common finding of anal SCC; the tumor has a predilection for metastasis to the mesorectal and internal iliac nodes above the pectinate line and the external iliac and inguinal lymph nodes below the demarcation.^16^

Most patients with anal cancer typically present with symptoms resembling benign diseases such as hemorrhoids.^5^ Patients commonly complain of rectal/abdominal pain and bleeding on toilet paper after wiping.^5,17^ In some cases, patients may present asymptomatic.^3^ In a study that surveyed 26 patients with newly diagnosed anal cancer, it was found that approximately 50% of patients never received a digital rectal examination (DRE) by their PCP, with 54% reporting having received no further evaluation upon the first visit to their physician and 27% receiving an inaccurate diagnosis of hemorrhoids at their first visit.^18^ This theme overlaps with the patient in our case as she was incorrectly diagnosed with hemorrhoids at her initial visit to her PCP. Her diagnosis was most likely missed due to a combination of low clinical suspicion and benign disease assumption. Diagnostic delays can be avoided by physicians keeping a high index of clinical suspicion in patients with rectal bleeding and pain. DREs should also be performed when symptoms of benign anal disease present upon clinical visits. Although cancers located higher up in the anal canal are less likely to cause symptoms and be found early, rectal examination done routinely may increase detection despite the low incidence of anal SCC.^19^ Additionally, patients at increased risk for AIN 2/3, which includes women with any history of cervical, vulvar, or vaginal cancer, men who have sex with men, solid organ transplant recipients, and immunocompromised due to any reason, should undergo screening with regular anal cytology, also known as anal pap testing.^4^ Routine screening is also recommended for all women who are HPV-16 positive and any patient over 35 years old with a history of HIV.^20,21^

## Conclusion

Anal cancer, a relatively rare disease, can cause abdominal discomfort, rectal pain, and bleeding in its early stages. The patient presented in this case report was misdiagnosed due to the similarities in the clinical presentation of anal cancer to benign diseases and low clinician cognizance. Patient education, in combination with physician awareness and suspicion, are essential ways to increase diagnostic accuracy. Although our patient had a negative HPV status, SCC of her anus may be associated with the infection, given its high-risk association with the malignancy and the possibility of a false negative test result. Patients similar to ours without known risk factors such as HPV and smoking add to the rarity of an already uncommon disease and further complicate the diagnosis. Physicians should retain a high index of clinical suspicion for anal cancer in patients with vague rectal symptoms such as mild abdominal discomfort, rectal pain, or bleeding. Therefore, early detection, proper examination, and prompt referral in conjunction with screening in high-risk patients may significantly reduce disease progression and patient discomfort.

## Funding

This case received no specific grant or support from any public or private agencies.

## Competing Interests

We declare no competing interests.

## Ethics

Ethical approval does not apply to this manuscript as it is a case report and not a case series. Written informed consent was obtained from the patient regarding the publication of this article and the associated image.

## Authors’ Contributions

Kevan wrote the article. Mercedes and Sandra reviewed and edited the manuscript. Dr. Semoin subsequently revised and made corrections to the article. All authors read and approved the final manuscript.
